# Development of conjugated secondary antibodies for wildlife disease surveillance

**DOI:** 10.3389/fimmu.2023.1221071

**Published:** 2023-07-11

**Authors:** Sunday O. Ochai, Jan E. Crafford, Pauline L. Kamath, Wendy C. Turner, Henriette van Heerden

**Affiliations:** ^1^Department of Veterinary Tropical Diseases, Faculty of Veterinary Science, University of Pretoria, Onderstepoort, South Africa; ^2^School of Food and Agriculture, University of Maine, Orono, ME, United States; ^3^U.S. Geological Survey, Wisconsin Cooperative Wildlife Research Unit, Department of Forest and Wildlife Ecology, University of Wisconsin-Madison, Madison, WI, United States

**Keywords:** wildlife species, adaptive immunity, avidity, conjugates, diagnostics, enzyme-linked immunosorbent assay (ELISA), passive disease surveillance

## Abstract

Disease monitoring in free-ranging wildlife is a challenge and often relies on passive surveillance. Alternatively, proactive surveillance that relies on the detection of specific antibodies could give more reliable and timely insight into disease presence and prevalence in a population, especially if the evidence of disease occurs below detection thresholds for passive surveillance. Primary binding assays, like the indirect ELISA for antibody detection in wildlife, are hampered by a lack of species-specific conjugates. In this study, we developed anti-kudu (*Tragelaphus strepsiceros*) and anti-impala (*Aepyceros melampus*) immunoglobulin-specific conjugates in chickens and compared them to the binding of commercially available protein-G and protein-AG conjugates, using an ELISA-based avidity index. The conjugates were evaluated for cross-reaction with sera from other wild herbivores to assess future use in ELISAs. The developed conjugates had a high avidity of >70% against kudu and impala sera. The commercial conjugates (protein-G and protein-AG) had significantly low relative avidity (<20%) against these species. Eighteen other wildlife species demonstrated cross-reactivity with a mean relative avidity of >50% with the impala and kudu conjugates and <40% with the commercial conjugates. These results demonstrate that species-specific conjugates are important tools for the development and validation of immunoassays in wildlife and for the surveillance of zoonotic agents along the livestock-wildlife-human interface.

## 1 Introduction

With the current increase in emerging and re-emerging disease outbreaks of both veterinary and human importance, there has been an urgent need for evidence-based methods for measuring both infection incidence and prevalence (1). Several techniques and interventions have been employed to mitigate the debilitating effects of disease-causing organisms on livestock and wildlife. However, what determines the choice of intervention to be implemented is the knowledge of the epidemiology governing or influencing these diseases (2). Wild animals are known to be hosts and/or reservoirs for pathogens that are of concern for cross-species transmission risk to humans and livestock. Therefore, an understanding of the epidemiology and ecology of pathogens in wildlife will better inform policies and interventions for control. Passive surveillance is currently used in most wildlife settings and can be largely dependent on the detection of clinical cases or case mortalities. However, opportunistic collection of mortality data and biases in the detection of carcasses and clinical signs can lead to distortion of the true incidence; therefore, a more active form of surveillance is needed (3). The detection of antibodies against pathogens can provide insights into prior exposure as well as information on the prevalence of a pathogen in an environment and the risk of pathogen spillover (3, 4). This approach may be especially useful for diseases with a short infection period, like anthrax, or those that do not cause mortality, like brucellosis.

Several serological techniques have been used to detect exposure to pathogens in African wildlife. These include primary binding assays like the enzyme-linked immunosorbent assay (ELISA) (5–7) as well as more historic secondary binding assays like the agar gel immunodiffusion (AGID) test and the complement fixation test (CFT) (8). Assays like the indirect ELISA can be highly sensitive and specific in the detection of pathogen-specific antibodies in the serum of a host, but they rely on a host-specific enzyme conjugate that limits the cross-species use of the assay. Many commercially available indirect ELISA kits are only validated for use in domestic animals. The enzyme-linked detection technique involves a highly specific antigen-antibody (Ag-Ab) interaction and was developed by Engvall and Perlmann (9). First, an antigen is restricted on a firm surface of a plate, followed by the addition of the sample antibody (if present), which then binds with a secondary antibody that is linked to an enzyme; next, this conjugated enzyme reaction is measured by incubation with a chromogen substrate (10). Horseradish peroxidase and alkaline phosphatase are the most used enzymes conjugated with secondary antibodies (11–13). Horseradish peroxidase used in this study has been demonstrated to bind effectively to immunoglobulins of mammalian and avian species (14). These conjugates, in a simpler sense, refer to an anti-species immunoglobulin that is linked to an enzyme that facilitates detection through color visualization. The ability to use conjugates of high avidity and specificity is therefore very important in measuring immune response using ELISA (15). The interaction and bond that exist between an antibody and an antigen are quite robust. The ability to be reversed and the strength of this bond are often dependent on the nature of the force that exists, which could be electrostatic, van der Waals, or hydrogen (16). Some of the binding forces are negatively associated with distance, and this makes them highly reliant on how well the molecules bind at the binding site (17). It is known that the measure of strength (affinity) of hapten-antibody binding (specific binding site) determines how well an antigen binds with an antibody (18). Avidity, on the other hand, is the total and cross-dependent binding strength of all the binding sites of an antibody to the multivalent antigen (18). It is therefore important to develop secondary antibodies that are of both high affinity and avidity. Antibody avidity is an important criterion for assessing immunological response. Furthermore, high-avidity antibodies perform better and may be more predictive of protective antibodies (19). Therefore, measuring the antibody avidity will give a better understanding of the functionality of antibodies and reduce the chances of false positive results in immunoassays. Species-specific conjugates for wildlife may not be available, and the generic conjugates that are used in these assays can vary significantly in binding to wildlife antibodies; results from these unvalidated assays should always be interpreted with caution (20–23).

Antibody avidity can be evaluated using ELISA in the presence of an immune-complex disruptive or disassociating compound like a chaotropic agent (18, 24–28). The thiocyanates can impact electrostatic interactions owing to their ionic characteristics, making them more widely acceptable (15, 29). There are a few reports about the use of different diluents for the chaotrope, including phosphate-buffered saline (PBS) (27, 30) and PBS + Tween (15, 28).

The paucity of studies around the use of ELISA for the surveillance of wildlife diseases is perhaps due to the lack or scarcity of species-specific conjugated secondary antibodies. There are various studies on the use of non-species-specific commercial conjugates such as protein A (protA), protein G (protG), and protein AG (pAG) for wildlife serological studies (15, 20, 22, 23, 31) (**Table S1**). Some commercial conjugates are available for domestic species (17) and some wildlife species, predominantly those from Europe (32). The variation in binding affinity for the commercial conjugates among various hosts shows that developing species-specific conjugates could be important in improving wildlife disease surveillance. Furthermore, the different methods used in these studies and the differences in data interpretation further complicate the synthesis of the results. Thus, it is important to develop conjugates that are specific to African wildlife and do not entirely rely on commercial multispecies conjugates.

Chickens have a greater immunological response to conserved mammalian proteins due to evolutionary distance. Egg yolk is an important alternative source of antibodies. In terms of productivity, animal welfare, and specificity, it outperforms mammalian serum immunoglobulins. Furthermore, because of structural differences and evolutionary distance, IgY is better suited for diagnostic purposes than mammalian antibodies, as it does not react with some components of the mammalian immune system and has a higher affinity for mammalian conserved proteins (19). The amount of antigen required for an effective immune response is relatively small (20-30 micrograms) (33). The use of complete Freund’s adjuvant results in long-lasting yolk antibody titers, yielding a total of 65 mg specific antibodies every month (19). Antibody purification is straightforward, affordable, and rapid as simple precipitation is adequate to achieve greater than 90% purity (33). In terms of animal welfare, daily collection of eggs is less stressful than daily bleeding of other laboratory animals. This makes the use of chickens as the source of antibody production efficient and better for the well-being of the laboratory species chosen (33). Therefore, developing polyclonal antibodies from chickens is relatively cheap and takes less time to produce compared to recombinant monoclonal antibodies (34). This becomes very important in resource-limited settings and under time constraints. Because polyclonal antibodies bind to more than a single epitope, their affinity is increased and makes them more tolerant to antigen changes (34).

Because wildlife hosts of pathogens of both veterinary and zoonotic importance are quite diverse globally, manufacturing species-specific conjugates for all host species seems impracticable; however, developing these for a few common hosts could improve disease surveillance efforts. In this study, we developed species-specific conjugates for kudu (*Tragelaphus strepsiceros*) and impala (*Aepyceros melampus*), respectively. These two species have been implicated as hosts for diseases like brucellosis (35, 36), anthrax (37, 38), and foot and mouth disease (39–41). We evaluated the binding avidity of these conjugates to several wildlife species and compared them to commercially available conjugates. We address the following questions (1): do developed novel species-specific conjugates for kudu and impala have better avidity than commercial conjugates? and (2) do these developed conjugates perform better across a range of related wildlife species? The validation of ELISA assays using conjugates specifically developed for pathogen detection in wildlife, rather than commercially available conjugates, is critical for improving wildlife disease surveillance and research.

## 2 Materials and methods

### 2.1 Experimental design and samples

Species-specific immunoglobulin conjugates for kudu and impala were developed by vaccinating Hyline Brown, Specific-Pathogen-Free (SPF) chickens (Avi-farms, Centurion, South Africa) with immunoglobulin (Ig) from kudu and impala (four animals per species), respectively. The chickens were raised in temperature-controlled pens, adhering strictly to the standard operating procedures of the Onderstepoort Veterinary Animal Research Unit of the Faculty of Veterinary Science, University of Pretoria. Anti-species immunoglobulin Y (IgY) were purified from egg yolks and conjugated to horseradish peroxidase. Cross-reactivity and avidity of the new conjugates were evaluated and compared to commercially available protein G (protG) and protein AG (pAG) conjugates using different herbivore species using an ELISA-based avidity index (AI). Serum samples from a variety of apparently healthy species (10 samples per species **Table 1**: for standardization purposes) were collected from South African National Parks (SANParks) biobanks and from samples banked in the Department of Veterinary Tropical Diseases, Faculty of Veterinary Science, University of Pretoria, South Africa. Species were classified into subfamilies and tribes based on Hassanin*, et al.* (42) and Gatesy*, et al.* (43) to give an indication of phylogenetic relatedness. Goat (*Capra hircus*), sheep (*Ovis aries*), and cattle (*Bos taurus*) samples were also included. Animal and research ethics from the University of Pretoria were obtained (REC063-19, REC041-19) and permits were obtained from the Department of Agriculture, Land Reform and Rural Development.

**Table 1:** List of species used for avidity and cross-reactivity tests.

Common Name	Species	Subfamily	Tribe
Greater kudu	*Tragelaphus strepsiceros*	Bovinae	Tragelaphini
Impala	*Aepyceros melampus*	Antilopinae	Aepycerotini
Burchell’s zebra	*Equus quagga burchellii*	Equinae	Equini
Black wildebeest	*Connochaetes gnou*	Alcelaphinae	Alcelaphini
African buffalo	*Syncerus caffer*	Bovinae	Bovini
Giraffe	*Giraffa camelopardalis*	Giraffinae	Giraffini
Blue wildebeest	*Connochaetes taurinus*	Alcelaphinae	Alcelaphini
Nyala	*Tragelaphus angasii*	Bovinae	Tragelaphini
Sable antelope	*Hippotragus niger*	Antilopinae	Hippotragini
Waterbuck	*Kobus ellipsiprymnus*	Hippotraginae	Hippotragini
Gemsbok	*Oryx gazella*	Antilopinae	Hippotragini
Springbok	*Antidorcas marsupialis*	Antilopinae	Antilopini
Hartebeest	*Alcelaphus buselaphus*	Antilopinae	Alcelaphini
Roan antelope	*Hippotragus equinus*	Antilopinae	Hippotragini
Common eland	*Taurotragus oryx*	Bovinae	Tragelaphini
Common tsessebe	*Damaliscus lunatus*	Antilopinae	Alcelaphini
Blesbok	*Damaliscus pygargus phillipsi*	Antilopinae	Alcelaphini
Bushbuck	*Tragelaphus scriptus*	Bovinae	Tragelaphini
Bontebok	*Damaliscus pygargus*	Antilopinae	Alcelaphini
Domestic goat	*Capra hircus*	Caprinae	Caprini
Domestic sheep	*Ovis aries*	Caprinae	Caprini
Domestic cattle	*Bos taurus*	Bovinae	Bovini

Species subfamily and tribe are as described by Hassanin*, et al.* (42) and Gatesy*, et al.* (43).

### 2.2 Precipitation of kudu and impala immunoglobulins

**Figure 1:**
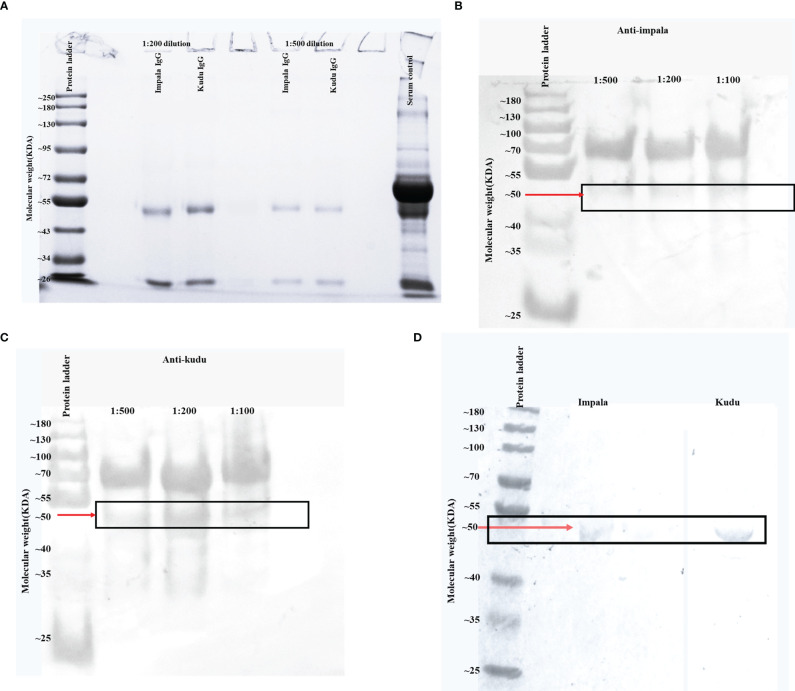
Images showing the required molecular weights of kudu and impala IgG as well as anti-kudu and anti-impala IgY binding to their respective IgG. **(A)** Sodium dodecyl-sulfate polyacrylamide gel electrophoresis (SDS-PAGE) image from the ammonium sulfate precipitated immunoglobulin fractions from kudu and impala sera. The protein bands at 50 and 25 KDa correspond to the heavy and light chains of IgG. Kudu serum was used as the serum control. **(B)** Western blot image indicating the binding of impala immunoglobulin G (IgG) to the chicken anti-impala IgY directly from theammonium sulfate precipitated egg yolk without affinity chromatography before conjugation. **(C)** Western blot image indicating the binding of kudu IgG to the chicken anti-kudu IgY directly from the ammonium sulfate precipitated egg yolk without affinity chromatography before conjugation. **(D)** Western blot image showing binding of impala (left) and kudu (right) IgG against the corresponding chicken affinity-purified IgY before conjugation. Red arrows with solid rectangles highlight the molecular weight of interest.

Immunoglobulin was extracted from kudu and impala by ammonium sulfate precipitation using the method described by Staak*, et al.* (44). Briefly, respective sera were diluted 1:4 with PBS (total volume 80 ml), while constantly stirring, 40 ml of saturated ammonium sulfate (Merck, Darmstadt, Germany) was slowly added to achieve a 33% saturation and the pH of the suspension was adjusted to 7.8 using a 2N NaOH (Associated Chemical Enterprises, Johannesburg, South Africa). The suspension was stirred continuously for 3 hours on a magnetic stirrer (Bibby Sterilin LTD, Staffordshire, England) and then centrifuged at room temperature for 30 minutes at 1400 × g using an Eppendorf centrifuge 5810R (Eppendorf, Hamburg, Germany), and the supernatant was discarded. The pellet was resuspended to a total volume of 80 ml in PBS and further purified by two additional cycles of precipitations, as described above. The final precipitate was dissolved in PBS in a volume half of the initial serum sample. Ammonium sulfate was removed by desalting spin columns (Thermo Scientific, Rockford, USA). IgG heavy and light chains were confirmed by SDS-PAGE gel electrophoresis (**Figure 1**).

The total protein concentration of the precipitated immunoglobulins (Ig) was determined using a spectrophotometer (Xpose™ Trinean Spectrophotometer, Trinean, Burladingen, Germany). The SDS-PAGE gel electrophoresis was performed as described by Laemmli (45) with a few modifications. Samples were diluted with the protein solvent buffer to a final concentration of 2 µg/µl. To determine the molecular size of the Ig, the protein was loaded into the wells of the SDS-PAGE at a concentration of 2 µg/µl. Samples were placed in Eppendorf tubes and put into a digital dry bath (Labnet Accublock Digital Dry Bath, Labnet International Inc, Woodbridge, USA) for 10 minutes at 100°C, after which they were spun using a mini centrifuge (Wealtec E-centrifuge, Wealtec corporation, Sparks, USA) for 10 seconds at 1400 × g. Gel reagents were mixed in volumes indicated in **Table S2**, and the solution was added between the clamped glass slides. The gel was allowed to polymerize for 30 minutes and then the stacking gel (**Table S2**) was added and incubated for 30 minutes. The gel was run at 100 V for 2 hours, after which it was stained with blue stain (GelCodeTM Blue stain, Thermo Scientific™, Massachusetts, USA). After the washing steps, the gel was viewed on a transilluminator (Univetec Cambridge transilluminator, Univetec, Cambridge, UK) for the presence of bands. Subsequently, the gel was transferred to the molecular image gel document system (Bio-rad molecular image gel document system, Bio-rad, California, USA) using the Image Lab software for analysis.

### 2.3 Immunization of chickens and extraction of IgY from eggs

Preparation of vaccines for immunizing chickens and extraction of IgY from egg yolk was adapted with modifications from Staak*, et al.* (44). Preparations of purified Ig from kudu and impala were made up to 200 µg/ml (w/v) in PBS. Then, 1 ml of vaccine (100 µg/ml) was prepared by emulsifying equal volumes (0.5 ml) of protein and Montanide ISA 50 V 2 adjuvant (SEPPIC, Paris, France) which was injected into both sides of the breast muscles. Inoculation was performed on Days 0, 23, and 42 (**Figure S1**). During this period, the development of specific IgY was monitored by testing the yolks in an ELISA (see antibody tires and method in **Supplementary Methodology 1** and **Figure S2**).

Egg yolks representing peak levels of anti-kudu or anti-impala IgY were harvested by separating the yolk from the albumin and diluting the yolk to 1:5 in distilled water before freezing at -20 °C for 72 hours. The suspension was thawed slowly at 4°C and centrifuged at 2800 x g for 20 minutes and the supernatant was collected. Ammonium sulfate was added in a concentration of 0.27g per ml of the supernatant and stirred for two hours at room temperature. Afterward, it was centrifuged at 2800 x g for 20 minutes and the supernatant was discarded. The pellet was resuspended in 24 ml of 2 M ammonium sulfate and stirred for 40 minutes at room temperature, followed by centrifugation and removal of the supernatant, as before. The precipitate was resuspended in 2.5 ml of PBS for each egg yolk and dialyzed against PBS at 4°C for 48 hours. Finally, the concentration of the immunoglobulin solution was measured and stored at -20°C (**Figure S1**).

Affinity chromatography using the polystyrene granulate method, as described by Staak*, et al.* (44) was used to further purify the recovered IgY. Briefly, 150 mg of impala and kudu IgG were immobilized separately on the granulated polystyrene using 0.05M carbonate buffer (pH 9.6), and free binding sites on the matrix were blocked using the blocking buffer (PBS; 0.005% Tween 20, PBST). Subsequently, the packed columns were equilibrated using PBST, and the chicken IgY were run through the columns using very slow rates to allow for optimal binding. Specific IgY were eluted by means of a glycine/hydrochloric acid elution buffer with a pH of 2.5. The affinity-purified IgY containing the light chains were used for the final conjugation.

A western blot was used to confirm the specificity of IgY produced against the respective Ig of kudu and impala. The western blot was performed as described by Howell, et al. (46), before and after affinity purification.

### 2.4 Horseradish peroxidase conjugation to IgY

The periodate method, as described by Wilson and Nakane (1978) and adapted by Staak*, et al.* (44), was used to conjugate the horseradish peroxidase (HRPO) to IgY. The activity of the conjugate was tested using a checkerboard titration between the kudu or impala serum, respectively (**Supplementary Methodology 2** and **Figure S2**, **Figure 1**).

### 2.5 Avidity index for cross-reactions between different conjugates and wildlife sera

The respective AIs for the binding of anti-kudu IgY and anti-impala IgY conjugates to kudu and impala sera as well as to the sera of the species listed in **Table 1** were compared. The binding of all the sera to protG- and pAG conjugates were also compared as described by Smit (15).

Briefly, a direct ELISA was employed by coating each microtiter plate (Thermo Scientific™ Pierce 96-well Plates-Corner, USA) with 10 sera samples per species at a dilution of 1:2000. Each plate was coated by adding 50 µl of the serum diluted in PBS in rows A-D of columns 1-10 for the 10 individual animals of the same species. Rows E-H of columns 1-10 were similarly filled with 50 µl of the next 10 sera of the second species. Columns 11 and 12 were filled with 50 µl of the control serum (kudu serum for anti-kudu conjugates, impala serum for anti-impala conjugates, cattle serum for pAG (15, 47), and goat serum for protG (48) at a concentration of 1:2000. Following incubation at 37°C for 1 hour on an orbital shaker, the plates were washed twice with PBS supplemented with 0.05% Tween-20 (PBST; Thermo Fisher Scientific, Waltham, MA USA) using a plate washer (Bio-Rad PW40, Mamesla-Coquette, France). Subsequently, all wells were loaded with PBST supplemented with 5% skimmed milk powder as a blocking step for 30 minutes at 37°C, and afterward, the wells were washed twice. The conjugates were diluted with PBSTM at a final concentration of 1:400 (as determined in **Supplementary Methodology 2** and **Figure S2**) for anti-kudu IgY and anti-impala IgY HRPO, and 1:10000 for pAG and protG as prescribed by the manufacturer. Afterward, 50 µl of PBS was added into the wells of rows A, B, E, and F, and rows C, D, G, and H were loaded with potassium thiocyanate as a chaotropic agent (CT) at a final concentration of 0.25 M. The plates were incubated for 1 hour at 37°C on the shaker and followed by a wash step. Color was developed by the addition of the ABTS substrate (2,2’-Azinobis [3-ethylbenzothiazoline-6-sulfonic acid]-diammonium salt; Thermo Scientific 1-Step ABTS, USA) and incubated in the dark for 30 minutes. The absorbance was read at 405 nm using the plate reader (Biotek Powerwave XS2, Vermont, USA) (**Figure 1**).

The avidity between the conjugate and serum was calculated as the reduction in color between wells without CT and those with CT and presented as the AI for each serum. AI was calculated as the mean ELISA absorbance values (ODs) from wells treated with the dissociating chaotrope (NH_4_SCN) divided by the mean ODs from wells without chaotrope and multiplied by 100.

### 2.6 Statistical analysis

To present the differences between the developed species-specific conjugates for kudu and impala and the commercial conjugates, we calculated the mean and standard deviation for the ODs and AI for both kudu and impala. A t-test was performed to measure the differences in the means of the ODs and AI for both the test samples and the controls as well as the performance of the IgY conjugates in the presence and absence of a chaotrope. The AI was defined as the ratio of both the OD of the CT-treated wells and the PBS-treated wells; the AI was calculated for each species and conjugate. The AI values for all species were normalized by subtracting them from the AI of their corresponding controls in order to measure how they differed from the respective control. A multivariate generalized linear model coupled with a Tukey’s Honestly Significant Difference (HSD) test for multiple mean comparisons was performed to compare the relationships between the AIs of the conjugates for the subfamily and tribes of the different species. The predictor variables included an interaction between conjugates and the subfamily and between conjugates and tribes, while the response variable was proportion (0-1) of the AI. All statistical analyses were done in R Console version 3.2.1 (49) with significance assessed using a threshold of alpha <0.05.

## 3 Results

### 3.1 Ammonium sulfate precipitation of IgG from Kudu and Impala Sera

The SDS-PAGE analysis confirmed the presence of two protein bands with molecular weights of around 50 and 25 KDa (for both kudu and impala) representing the heavy and light chains of IgG (**Figure 1A**).

### 3.2 Western blot

The western blot analysis confirmed the specificity of the IgY against the IgG of impala (**Figure 1B**) and kudu (**Figure 1C**). **Figures 1B, C** (before affinity chromatography) and Figure D (after affinity chromatography) show the specificity of the immunoglobulins produced. Only binding to the 50 KDa heavy chain, which is the primary component of the Fc portion, was observed to confirm the specificity of the secondary antibodies.

### 3.3 Binding activities of anti-kudu IgY, anti-impala IgY, and commercial conjugates on kudu and impala sera

**Figure 2:**
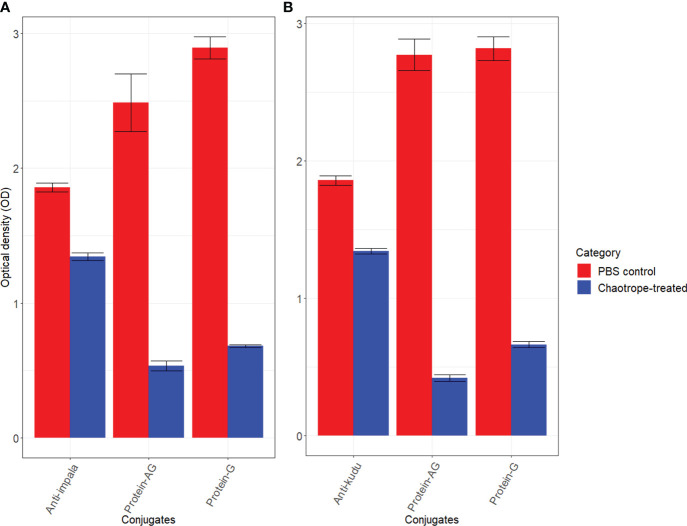
Bar charts with error bars (standard deviation) showing the differences in mean optical densities (OD) for the developed and commercial conjugates: **(A)** impala sera against anti-impala IgY, protein AG, and protein G conjugates, and **(B)** kudu sera against anti-kudu IgY, protein AG and protein G conjugates. The red bars represent wells without the chaotrope and the blue bars represent wells that received dissociating chaotrope. For each species, 10 replicates were used for the experiments.

Kudu and impala sera bound significantly better with their respective conjugates compared to the commercial conjugates (*p*<0.0001). There was also a significant drop in optical densities for the commercial conjugates in the presence of the chaotrope (*p*<0.0001) but not the developed conjugates (*p*>0.05; **Figure 2**). For the anti-kudu IgY conjugate on kudu serum, the mean AI was 72.36 ± 1.13 SD, compared to 15.23 ± 1.1 SD for pAG and 23.61 ± 0.99 SD for protG. For anti-impala IgY conjugate on impala serum, the mean AI was 72.09 ± 0.89 SD, compared to 21.47 ± 0.66 SD for pAG and 23.52 ± 0.56 SD for protG.

### 3.4 Binding activities of anti-kudu IgY, anti-impala IgY, and commercial conjugates on kudu and impala sera

**Figure 3:**
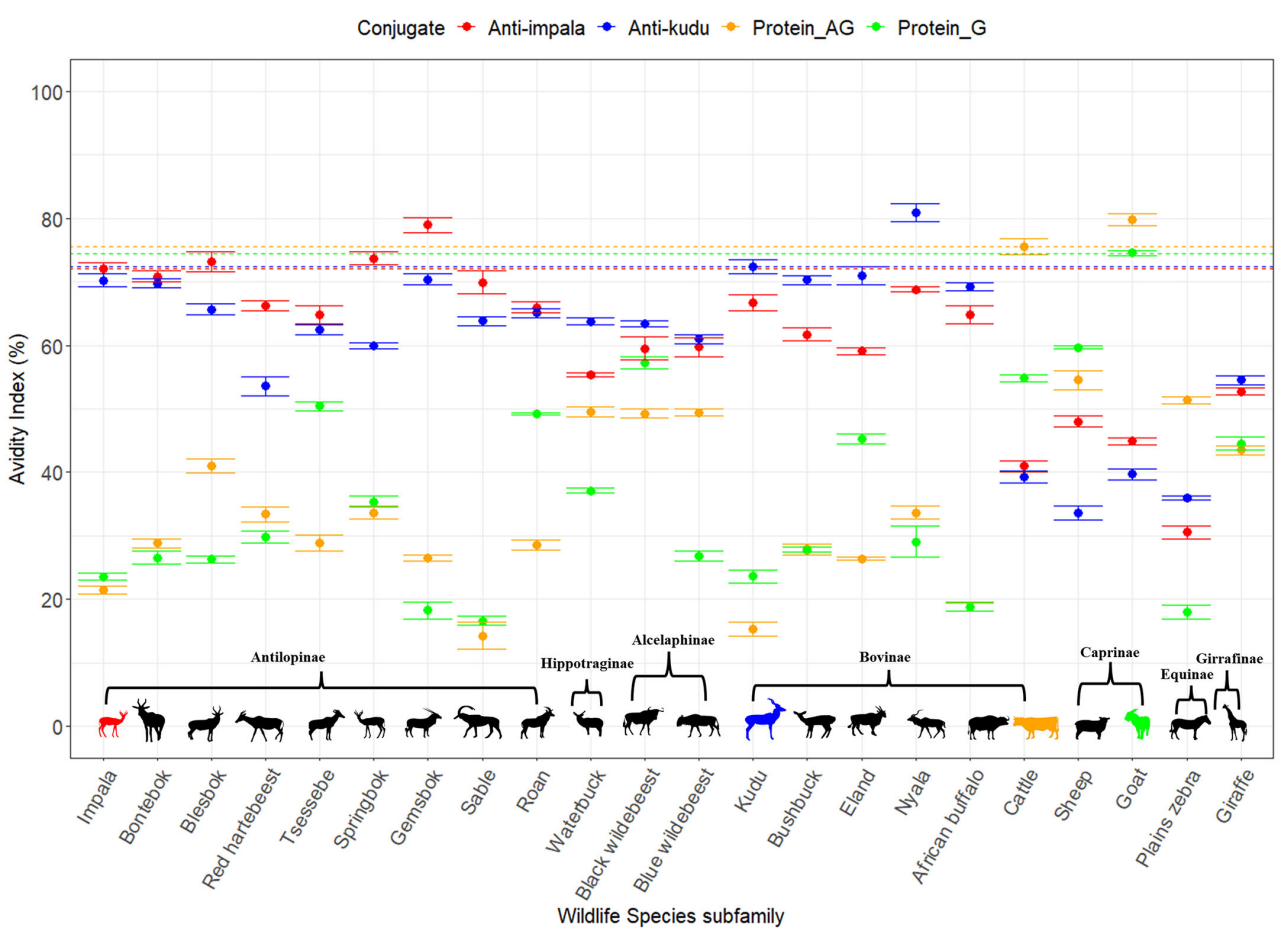
Scatter plot with error bars (standard deviation) showing the avidity index for each of the conjugates (red anti-impala, blue anti-kudu, yellow protein AG, and green protein G) determined for different wildlife species (**Table 1**). The avidity between the conjugate and different sera was calculated as the reduction in color between wells without a chaotropic agent (CT) and those with CT and presented as the AI for each serum. The silhouettes in color connect species and conjugate colors to denote the species used as a control for each conjugate: impala for anti-impala IgY, kudu for anti-kudu IgY, cattle for protein AG, and goat for protein G. The horizontal dotted lines indicate the avidity index of the respective controls, and the colors correspond to the conjugates. Species were grouped into subfamilies as described by Hassanin, et al. (38); however, ordering of the species was not done by phylogenetic relationships.

**Figure 4:**
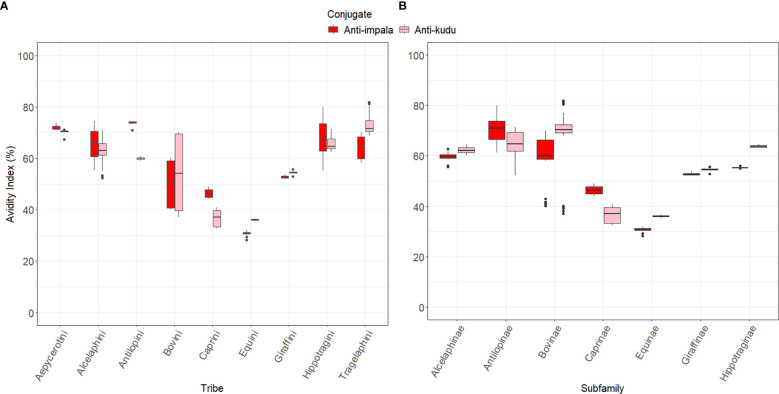
Box plots showing the avidity index for the wildlife species grouped by **(A)** the tribe they belong to and **(B)** their subfamilies. These species were classified based on the work described by Hassanin, et al. (38) and Gatesy, et al. (39). Red indicates anti-impala IgY and pink is anti-kudu IgY conjugate.

When comparing how each host species reacted to the conjugates, we found that kudu serum had mean AIs of 72.36 ± 1.07 with anti-kudu IgY and 66.67 ± 1.17 with anti-impala IgY. There was a significant difference between the anti-kudu IgY, anti-impala IgY, pAG, and protG conjugates for kudu sera (*p*<0.0001; **Figure 3**). Similarly, impala serum had AIs of 72.08 ± 0.88 with anti-impala IgY, 70.20 ± 0.99 with anti-kudu IgY, 21.47 ± 0.62 with pAG, and 23.52 ± 0.53 with protG conjugates (**Figure 4**). There was also a significant difference among all the conjugates for impala sera (*p*<0.05).

Our developed IgY conjugates out-performed the commercial conjugates for all wildlife species except for zebra specifically, with an AI of less than 50 (anti-kudu IgY=30.54 ± 1.04; anti-impala IgY= 35.97 ± 0.37). The average AI for anti-impala IgY across all the species was 61.73 ± 11.25 (**Table S3**); for anti-kudu IgY, it was 63.25 ± 11.51 (**Table S4**); for pAG, it was 37.71 ± 17.25 (**Table S5**); and for protG, it was 36.08 ± 15.78 (**Table S6**). All wildlife sera tested with the protG conjugate had an AI of less than 50, except for black wildebeest (57.24 ± 0.88) and tsessebe (50.38 ± 0.64) (**Figure 3**). Also, all the wildlife sera that were tested with the pAG conjugate demonstrated an AI of less than 50%, except for plains zebra (51.35 ± 0.48). The individual AIs for the wildlife sera are shown in **Figure 3**.

There were significant differences (*p*<0.05) between each species and its respective controls, except for impala and blesbok (p=0.088; **Table S3**). All the animals had an avidity index below the respective controls, except for gemsbok and nyala, which were higher than kudu (anti-kudu); goat, which was higher than cattle (pAG); and springbok, which was higher than impala (**Figure 3**). Details of all the normalized AIs are shown in **Table S3**. Values above the zero threshold indicate higher avidity than the respective control, while negative values indicate lower avidity compared to the control. Comparing the differences in avidity of the developed conjugates to the different wildlife species, there was a significant interaction between the developed conjugates (anti-impala IgY and anti-kudu Ig conjugates) and the subfamily of the wildlife species (*p*<0.0001; **Figures 4A, B**). Antilopinae and Caprinae subfamilies did significantly better with anti-impala, while the Bovinae, Alcelaphinae, and Hippotraginae subfamilies did better with anti-kudu (p<0.0001; **Figures 4A, B**). Tribes and subfamilies more closely related to kudu performed better with anti-kudu conjugate than anti-impala, and wildlife species more closely related to impala performed better with anti-impala. There was a wider variation in tribes than in subfamilies, as shown in **Figure 4A**. Animals that share the same tribe, such as domestic cattle and the African buffalo demonstrated significant variation (p<0.0001) in their avidity to both the commercial and developed conjugate. Domestic cattle performed significantly better with pAG and protG than the African buffalo, while the African buffalo demonstrated better avidity to the developed conjugates than domestic cattle.

## 4 Discussion

We developed conjugates for kudu (IgY anti-kudu) and impala (IgY anti-impala), two important hosts in disease transmission in wildlife in South Africa. This study shows that the conjugates are specific to their respective species and have better avidity than the commercially available protG and pAG conjugates. This is the first study to develop species-specific conjugates for antibody detection in kudu and impala with quantitative evidence of cross-reactions with antibodies of other species of African wildlife, providing the tools for the development and validation of primary binding assays like the indirect ELISA. These assays can improve sero-surveillance for infectious diseases in wildlife.

Chicken anti-kudu and chicken anti-impala conjugates developed in this study confirm that the benefit of using IgY in developing secondary antibodies against mammalian sera IgY from eggs. These conjugates are cheap to produce in large volumes and ethically preferable as no blood collection from animals is required (50). This study shows that the developed anti-kudu IgY and anti-impala IgY conjugates had higher AIs (>70%) compared to commercial pAG and protG conjugates with AIs less than 30%. This confirms stronger binding of the secondary IgY antibodies, which is an important parameter in the development of primary binding assays like the indirect ELISA (27). The weak binding observed for protG and pAG conjugates to impala sera in this study was also observed in other studies (15, 22, 23, 31) and is also in agreement with the findings of Smit (15). However, it contradicts other findings that indicate strong reactivity with either protG or pAG (20, 22, 23, 31). This could be due to the differences in the methods used. In this study, we measured the binding strength of the antibodies in the presence of a dissociative agent, whereas other studies only compared the binding of conjugates under normal physiological conditions. Similar to this study, Smit (15) also recorded high OD values for protA and pAG but showed that the avidity was weak and binding could easily be disrupted under stringent binding conditions, like in the presence of the chaotropic agent.

Sera from the different species reacted differently with the two developed IgY conjugates and the two commercial conjugates. Wildlife species had stronger binding to the IgY conjugates than to the commercial conjugates, except for the plains zebra. Although the wildlife species demonstrated good avidity with both anti-kudu IgY and anti-impala IgY conjugates, there appeared to be a phylogenetic basis for performance of the two IgY conjugates. The antelopes more closely related to kudu had better avidity to the anti-kudu IgY conjugate and those more closely related to impala had better avidity to the anti-impala IgY conjugate (**Figures 3**). Species-specific conjugates can also bind with good avidity to closely related species (15). This means that the more distantly related they are to the species for which the conjugate was developed, the lower their avidity. For example, sable, roan, tsessebe, blesbok, and bontebok (see **Table 1** for scientific names) had better avidity with anti-impala IgY as they all belong to the *Antilopinae* family, as described by Hassanin*, et al.* (42). Similarly, the members of the Tragelaphini tribe, such as nyala, eland, and bushbuck (see **Table 1** for scientific names) (42) had better avidity with the anti-kudu IgY conjugate (**Figures 3**, **4A, B**). A weaker avidity was seen in more distantly related species like cattle, goats, plains zebra, and giraffes (see **Table 1** for scientific names; **Figures 3**, **4**).

There are reports in the literature of assays developed for livestock being used for antibody detection in wildlife. These include studies in which assays developed for horses were used for zebra (51), assays developed for domestic dogs were used for African wild dogs (52), assays developed for domestic cats were used for lions (53), and assays developed for domestic cattle were used for African buffalo (54). However, in this study, we report a significant variation between domestic cattle and African buffalo within the Bovini tribe. African buffalo reacted strongly with anti-kudu and anti-impala conjugates, with an avidity of greater than 60%, but had a poor avidity of less than 20% with pAG and protG conjugates. Domestic cattle, on the other hand, had a stronger avidity with pAG and protG conjugates but demonstrated a poor avidity index with anti-kudu and anti-impala conjugates (≤40%). These results emphasize the need to develop and validate serological assays that are specific for wildlife species and caution against interspecies use of assays without proper validation, even if they belong to the same tribes.

The conjugates developed here are important tools for the development of validated assays for the surveillance of emerging and re-emerging diseases of veterinary and human importance. Furthermore, the concept of a diagnostic test being fit and validated for specific host species is one that is critical and promoted by the World Organization for Animal Health (WOAH, founded as OIE) (55). Wildlife diseases are often understudied, and little is known about the accuracy of the diagnostic techniques employed (56). One pertinent question that remains is about the accuracy of the diagnostic tests validated in domestic stocks when used in wildlife species. The majority of the wild animals tested in this study are important hosts to various pathogens responsible for a range of diseases in the wild. These animals demonstrated strong avidity with either anti-impala or anti-kudu; this is an indication that developing a multi-species polyclonal conjugate consisting of a cocktail of immunoglobulins could further improve active surveillance and facilitate the validation of immunoassays in these species. Although these conjugates were developed and evaluated for wildlife species, the idea of developing conjugates along phylogenetic lines may provide solutions in other locations, such as Amazônia and the Cerrado biome in Brazil, experiencing similar challenges (57).

The pAG conjugate tested in this study demonstrated an avidity index of less than 40% with most wildlife species, with the exception of the plains zebra, black and blue wildebeest, and waterbuck. These results corroborate the findings of Smit (15), who reported similar AIs in these species. The poor reactivity seen in the majority of the wildlife species could be attributed to a genetic predisposition that could make pAG bind weakly with the IgG of the wildlife species. Except for black wildebeest and tsessebe, all the wildlife species had an AI of less than 50% with the protG conjugate used in this study. Factors that could influence the binding of conjugates in primary binding assays could include variation in antibody structure between species, a limited amount of IgG in the original serum, as seen in immunocompromised individuals, or the presence of inhibitors (20, 23). Also, pAG and protG could selectively bind to the subclasses or isotypes of IgG, as seen in mice, where IgG2 is bound more strongly to protG, while IgG1 binds very weakly (58). Therefore, when an immune response is predominantly of a different subclass, these subclasses may not be detected in an immunoassay that is utilizing these conjugates. The variation in the avidity of conjugates to the immunoglobulins of different species emphasizes the importance of proper species-specific validation of diagnostic assays.

## 5 Conclusion

The results of this study demonstrate the need to develop conjugates for immunoassays that are specific to African wildlife as they are important hosts to many pathogens of human, animal, and zoonotic importance in KNP and parks like it. Kudu and impala sera demonstrated better avidity to their corresponding conjugates than to the commercial conjugates. The wildlife species tested in this study showed stronger avidity to the developed conjugates than to the commercial conjugates. This stronger avidity could also be achieved through a multi-species polyclonal conjugate consisting of a cocktail of immunoglobulins from various wildlife species. Such evidence-based methods could allow for more accurate validation of diagnostic assays for the detection of the incidence and prevalence of wildlife and zoonotic diseases.

## 6 Suggestions for future research

Future studies to examine the development of polyclonal cocktail conjugated secondary antibodies for other African wildlife could establish immunodiagnostic assays that would be more specific to identify pathogens of veterinary and human diseases. Secondly, owing to the varying reports of avidity and binding ability of commercial conjugates, we suggest studies that evaluate these conjugates on a wider selection of wildlife species beyond what is covered in this study. Even though this study was conducted on African wildlife, we suggest further studies focused on how the use of different conjugates affects the outcome of disease surveillance and screening in other settings outside Africa.

## Data availability statement

The original contributions presented in the study are included in the article/**Supplementary Material**. Further inquiries can be directed to the corresponding author.

## Ethics statement

The animal study was reviewed and approved by Faculty of Veterinary Science Animal Ethics Committee, University of Pretoria.

## Author contributions

SO and HH conceived the ideas for the study. SO, HH, and JC designed the study. SO collected the data. SO, JC, and HH designed the methodology. SO analyzed the data. SO and HH wrote the first draft of the manuscript. All authors contributed significantly to the manuscript revision and read and gave approval for publication.

## References

[1] Lambert S Gilot-Fromont E Toïgo C Marchand P Petit E Rossi S. Combining seroprevalence and capture-mark-recapture data to estimate the force of infection of brucellosis in a managed population of alpine ibex. Epidemics (2022) 38:100542. doi: 10.1016/j.epidem.2022.100542 35152060

[2] Artois M Bengis R Delahay RJ Duchêne M-J Duff JP Ferroglio E. Wildlife disease surveillance and monitoring. Springer Japan; (2009) p:187–213. doi: 10.1007/978-4-431-77134-0_10

[3] Garnier R Ramos R Sanz-Aguilar A Poisbleau M Weimerskirch H Burthe S. Interpreting ELISA analyses from wild animal samples: some recurrent issues and solutions. Funct Ecol (2017) 31(12):2255–62. doi: 10.1111/1365-2435.12942

[4] Gardner IA Hietala S Boyce WM. Validity of using serological tests for diagnosis of diseases in wild animals. Rev scientifique technique. (1996) 15(1):323–35. doi: 10.20506/rst.15.1.926 8924713

[5] Kock ND Jongejan F Kock MD Kock RA Morkel P. Serological evidence for cowdria ruminantium infection in free-ranging black (Diceros bicornis) and white (Ceratotherium simum) rhinoceroses in Zimbabwe. J Zoo Wildlife Med (1992) 23(4):409–13.

[6] Turnbull PC Doganay M Lindeque PM Aygen B McLaughlin J. Serology and anthrax in humans, livestock and etosha national park wildlife. Epidemiol infect (1992) 108(2):299–313. doi: 10.1017/S0950268800049773 1582472 PMC2271992

[7] Lembo T Hampson K Auty H Beesley CA Bessell P Packer C. Serologic surveillance of anthrax in the Serengeti ecosystem, Tanzania, 1996-2009. Emerg Infect dis (2011) 17(3):387–94. doi: 10.3201/eid1703.101290 PMC3166018 21392428

[8] Blackburn NK Swanepoel R. Observations on antibody levels associated with active and passive immunity to African horse sickness. Trop Anim Health Product (1988) 20(4):203–10. doi: 10.1007/BF02239981 3238767

[9] Engvall E Perlmann P. Enzyme-linked immunosorbent assay (ELISA). Quantitat assay immunoglobulin G. Immunochemistry. (1971) 8(9):871–4. doi: 10.1016/0019-2791(71)90454-x 5135623

[10] ThermoFisher scientific overview of ELISA - ZA (2019). Available at: https://www.thermofisher.com/za/en/home/life-science/protein-biology/protein-biology-learning-center/protein-biology-resource-library/pierce-protein-methods/overview-elisa.html.

[11] Voller A Bidwell D Huldt G Engvall E. A microplate method of enzyme-linked immunosorbent assay and its application to malaria. Bull World Health Organ. (1974) 51(2):209–11. PMC2366227 4377238

[12] Payment P Descoteaux JP. Enzyme-linked immunosorbent assay for the detection of antibodies to pneumonia virus of mice in rat sera. Lab Anim science. (1978) 28(6):676–9. 374862

[13] Rennard SI Berg R Martin GR Foidart JM Robey PG. Enzyme-linked immunoassay (ELISA) for connective tissue components. Anal Biochem (1980) 104(1):205–14. doi: 10.1016/0003-2697(80)90300-0 6992646

[14] Wilson MB Nakane P. P. Recent developments in the periodate method of conjugating horse radish peroxidase (HRPO) to antibodies, inImmunofluorescence and Related Staining Techniques. (Knapp W. Holubar K. Wick G., eds.), Elsevier/North Holland Biomedical, Amsterdam, pp. 215–224.

[15] Smit SA. Evaluation of anti-bovine, anti-equine and recombinant protein A/G horseradish peroxidase conjugates for cross reactivity to wildlife serum antibodies using ELISA. University of Pretoria (2017).

[16] van Oss CJ Chaudhury MK Good RJ. Monopolar surfaces. Adv Colloid Interface Science. (1987) 28:35–64. doi: 10.1016/0001-8686(87)80008-8 3333137

[17] BioRad. Helpful ELISA hints | online: @BioRadAbs (2021). Available at: https://www.bio-rad-antibodies.com/helpful-elisa-hints.html.

[18] Hudson L Hay FC Hudson L. Practical immunology. Oxford: Blackwell Scientific Publications (1989).

[19] Gassmann M Thömmes P Weiser T Hübscher U. Efficient production of chicken egg yolk antibodies against a conserved mammalian protein. FASEB J (1990) 4(8):2528–32. doi: 10.1096/fasebj.4.8.1970792 1970792

[20] Kelly PJ Tagwira M Matthewman L Mason PR Wright EP. Reactions of sera from laboratory, domestic and wild animals in Africa with protein a and a recombinant chimeric protein AG. Comp immunology Microbiol Infect dis (1993) 16(4):299–305. doi: 10.1016/0147-9571(93)90159-3 8281743

[21] Pruvot M Forde T Steele J Kutz S De Buck J van der Meer F. The modification and evaluation of an ELISA test for the surveillance of mycobacterium avium subsp. paratuberculosis infection Wild ruminants. BMC veterinary Res (2013) 9:5. doi: 10.1186/1746-6148-9-5 PMC3545983 23302439

[22] Stoübel K Schoünberg A Staak C. A new non-species dependent ELISA for detection of antibodies to borrelia burgdorferi s. l. in zoo animals. Int J Med Microbiol (2002) 291(Supplement 33):88–99. doi: 10.1016/S1438-4221(02)80018-2 12141767

[23] Kramsky JA Manning EJ Collins MT. Protein G binding to enriched serum immunoglobulin from nondomestic hoofstock species. J Veterinary Diagn Invest (2003) 15(3):253–61. doi: 10.1177/104063870301500306 12735347

[24] Hedman K Seppälä I. Recent rubella virus infection indicated by a low avidity of specific IgG. J Clin Immunol (1988) 8(3):214–21. doi: 10.1007/BF00917569 3292566

[25] MacDonald RA Hosking CS Jones CL. The measurement of relative antibody affinity by ELISA using thiocyanate elution. J Immunol Methods (1988) 106(2):191–4. doi: 10.1016/0022-1759(88)90196-2 3339255

[26] Westerlund A Ankelo M Ilonen J Knip M Simell O Hinkkanen AE. Absence of avidity maturation of autoantibodies to the protein tyrosine phosphatase-like IA-2 molecule and glutamic acid decarboxylase (GAD65) during progression to type 1 diabetes. J Autoimmun (2005) 24(2):153–67. doi: 10.1016/j.jaut.2004.12.001 15829408

[27] Dimitrov JD Lacroix-Desmazes S Kaveri SV. Important parameters for evaluation of antibody avidity by immunosorbent assay. Analyt Biochem (2011) 418(1):149–51. doi: 10.1016/j.ab.2011.07.007 21803020

[28] Dauner JG Pan Y Hildesheim A Kemp TJ Porras C Pinto LA. Development and application of a GuHCl-modified ELISA to measure the avidity of anti-HPV L1 VLP antibodies in vaccinated individuals. Mol Cell Probes. (2012) 26(2):73–80. doi: 10.1016/j.mcp.2012.01.002 22285687 PMC3319198

[29] Almanzar G Ottensmeier B Liese J Prelog M. Assessment of IgG avidity against pertussis toxin and filamentous hemagglutinin via an adapted enzyme-linked immunosorbent assay (ELISA) using ammonium thiocyanate. J Immunol Methods (2013) 387(1-2):36–42. doi: 10.1016/j.jim.2012.09.008 23022630

[30] Ferreira MU Katzin AM. The assessment of antibody affinity distribution by thiocyanate elution: a simple dose-response approach. J Immunol Methods (1995) 187(2):297–305. doi: 10.1016/0022-1759(95)00186-4 7499889

[31] Feir D Lau C Junge R. Protein a and protein G in the diagnosis of diseases in zoo animals. Trans Missouri Acad Science. (1993) 27:9–14.

[32] Rossi S Pioz M Beard E Durand B Gibert P Gauthier D. Bluetongue dynamics in French wildlife: exploring the driving forces. Transbound Emerg Dis (2014) 61(6):e12–24. doi: 10.1111/tbed.12061 23414427

[33] Gassmann M Weiser T Thömmes P Hübscher U. [The chicken egg as a supply of polyclonal antibodies]. Schweiz Arch Tierheilkd. (1990) 132(6):289–94. 2205000

[34] Pereira EPV van Tilburg MF Florean E Guedes MIF. Egg yolk antibodies (IgY) and their applications in human and veterinary health: a review. Int Immunopharmacol. (2019) 73:293–303. doi: 10.1016/j.intimp.2019.05.015 31128529 PMC7106195

[35] Godfroid J. Brucellosis in livestock and wildlife: zoonotic diseases without pandemic potential in need of innovative one health approaches. Arch Public Health (2017) 75:34. doi: 10.1186/s13690-017-0207-7 28904791 PMC5592711

[36] Simpson G Thompson PN Saegerman C Marcotty T Letesson J-J de Bolle X. Brucellosis in wildlife in Africa: a systematic review and meta-analysis. Sci Rep (2021) 11(1):5960. doi: 10.1038/s41598-021-85441-w 33727580 PMC7966391

[37] De-Vos V. The ecology of anthrax in the Kruger national park, south Africa. Salisbury Med Bulletin. (1990) 68S::19–23.

[38] Basson L Hassim A Dekker A Gilbert A Beyer W Rossouw J. Blowflies as vectors of bacillus anthracis in the Kruger national park. (2018) 60(1):. doi: 10.4102/koedoe.v60i1.1468

[39] Wittmann G. [Virus carriers in foot-and-mouth disease. review]. Berl Munch Tierarztl Wochenschr (1990) 103(5):145–50. 2191649

[40] Vosloo W Bastos A Sahle M Sangare O Dwarka R. Virus topotypes and the role of wildlife in foot and mouth disease in Africa. (2005), 67–73.

[41] Letshwenyo M Mapitse N Hyera J. Foot-and-mouth disease in a kudu (Tragelaphus strepsiceros) in Botswana. Veterinary Rec (2006) 159:252–3. doi: 10.1136/vr.159.8.252 16921016

[42] Hassanin A Emmanuel JPD. Evolutionary affinities of the enigmatic saola (Pseudoryx nghetinhensis) in the context of the molecular phylogeny of bovidae. Proceedings: Biol Sci (1999) 266(1422):893–900. doi: 10.1098/rspb.1999.0720 PMC1689916 10380679

[43] Gatesy J Amato G Vrba E Schaller G DeSalle R. A cladistic analysis of mitochondrial ribosomal DNA from the bovidae. Mol Phylogenet Evolution. (1997) 7(3):303–19. doi: 10.1006/mpev.1997.0402 9187090

[44] Staak C Salchow F Denzin N. Practical serology: from the basics to the testing. München: Urban & Vogel (2001).

[45] Laemmli UK. Cleavage of structural proteins during the assembly of the head of bacteriophage T4. Nature (1970) 227(5259):680–5. doi: 10.1038/227680a0 5432063

[46] Howell PG Groenewald D Visage CW Bosman AM Coetzer JA Guthrie AJ. The classification of seven serotypes of equine encephalosis virus and the prevalence of homologous antibody in horses in south Africa. Onderstepoort J Vet Res (2002) 69(1):79–93. 12092781

[47] Inoshima Y Shimizu S Minamoto N Hirai K Sentsui H. Use of protein AG in an enzyme-linked immunosorbent assay for screening for antibodies against parapoxvirus in wild animals in Japan. Clin Diagn Lab Immunol (1999) 6(3):388–91. doi: 10.1128/CDLI.6.3.388-391.1999 PMC103728 10225841

[48] ThermoFisher S. Pierce™ recombinant protein G 2023 . Available at: https://www.thermofisher.com/order/catalog/product/21193.

[49] R Core Team. R:A language and environment for statistical computing. Vienna, Austria: R Foundation for Statistical Computing (2017). Available at: http://www.R-project.org/.

[50] Amro WA Al-Qaisi W Al-Razem F. Production and purification of IgY antibodies from chicken egg yolk. J Genet Eng Biotechnol (2018) 16(1):99–103. doi: 10.1016/j.jgeb.2017.10.003 30647711 PMC6296578

[51] Abdelgawad A Hermes R Damiani A Lamglait B Czirják G East M. Comprehensive serology based on a peptide ELISA to assess the prevalence of closely related equine herpesviruses in zoo and wild animals. PloS One (2015) 10(9):e0138370. doi: 10.1371/journal.pone.0138370 26378452 PMC4574707

[52] Kat PW Alexander KA Smith JS Munson L. Rabies and African wild dogs in Kenya. Proceedings: Biol Sci (1995) 262(1364):229–33. 10.1098/rspb.1995.0200 8524915

[53] Gumbo R Sylvester TT Goosen WJ Buss PE de Klerk-Lorist LM van Schalkwyk OL. Adaptation and diagnostic potential of a commercial cat interferon gamma release assay for the detection of mycobacterium bovis infection in African lions (Panthera leo). Pathogens (2022) 11(7):765–73. doi: 10.3390/pathogens11070765 PMC9317741 35890010

[54] Sarangi LN Surendra KSNL Rana SK Naveena T Prasad A Ponnanna NM. Evaluation of commercial ELISA kits for diagnosis of brucellosis in cattle and buffaloes in different epidemiological scenarios. J Microbiol Methods (2022) 195:106449. doi: 10.1016/j.mimet.2022.106449 35318085

[55] Gardner IA Colling A Greiner M. Design, statistical analysis and reporting standards for test accuracy studies for infectious diseases in animals: progress, challenges and recommendations. Prev Vet Med (2019) 162:46–55. doi: 10.1016/j.prevetmed.2018.10.023 30621898

[56] Jia B Colling A Stallknecht DE Blehert D Bingham J Crossley B. Validation of laboratory tests for infectious diseases in wild mammals: review and recommendations. J Vet Diagn Invest. (2020) 32(6):776–92. doi: 10.1177/1040638720920346 PMC7649530 32468923

[57] Barbaru-Grajales A Peñafiel-Arcos PA Suarez-Cedillo SE Barreno-Silva NDeLM Acosta-Perez KI. Monitoring of diseases in wild plants and animals: a challenge to measure the success of environmental policies and management in Ecuador. Cent Eur Manage J (2023) 31(2):234–341. doi: 10.57030/23364890.cemj.31.2.27

[58] Björck L Kronvall G. Purification and some properties of streptococcal protein G, a novel IgG-binding reagent. J Immunol (1984) 133(2):969–74. doi: 10.4049/jimmunol.133.2.969 6234364

