# Supplementary material for: Development of conjugated secondary antibodies for wildlife disease surveillance
Source: Front Immunol. 2023 Jul 11;14:1221071. doi: 10.3389/fimmu.2023.1221071 (PMC10368751; doi:10.3389/fimmu.2023.1221071)
Supplement: Supplementary file 1 [file DataSheet_1.pdf]

Development of conjugated secondary antibodies for wildlife disease surveillance

Sunday O. Ochai[1*], Jan E. Crafford[1], Pauline L. Kamath [2,] Wendy C. Turner[3], Henriette van Heerden[1]

[1] Department of Veterinary Tropical Diseases, Faculty of Veterinary Science, University of Pretoria, Onderstepoort, South Africa.

[2] School of Food and Agriculture, University of Maine, Orono, Maine, U.S.A.

[3] U.S. Geological Survey, Wisconsin Cooperative Wildlife Research Unit, Department of Forest and Wildlife Ecology, University of Wisconsin-Madison, Madison, Wisconsin, U.S.A

* Correspondence: Sunday O. Ochai; s.o.ochaijr@gmail.com

Disclaimer: Any use of trade, product, or firm names is for descriptive purposes only and does not imply endorsement by the U.S. Government.

Supplementary information

Table S1: Summary of results obtained from publications that reported the use of commercially available Protein A, Protein G, and Protein AG in some African wildlife species and domestic stock. The table contains references of publications, methods used in parenthesis, and different interpretations of results. NA here stands Not Applicable

		**Kelly, *et al.*, (1) (Direct ELISA)**		**Stöbel, *et al.*, (2) (Indirect ELISA)**		**Feir, *et al.*, (3) (Indirect ELISA)**		**Kramsky, *et al.*, (4)* (Direct ELISA)**	**Smit (5) (Direct Avidity ELISA)**
**Species**	**Common name**	**Protein A**	**Protein AG**	**Protein A**	**Protein G**	**Protein A**	**Protein G**	**Protein G**	**Protein AG**
Loxodonta africana	African elephant	Weak	Weak	Medium	Low	NA	NA		Weak
Giraffa camelopardalis	Giraffe	Weak	Weak	Low	High	NA	NA		Weak
Aepyceros melampus	Impala	Weak	Strong	None	Low	None	None	Lesser to control	No reaction
Connochaetes taurinus	Blue wildebeest	Weak	Strong	NA	NA	NA	NA		Moderate
Syncerus caffer	African buffalo	Weak	Strong	None	Low	NA	NA		Strong
Tragelaphus angasii	Nyala	Weak	Strong	None	Low	NA	NA		Weak
Taurotragus oryx	Common eland	Weak	Strong	NA	NA	NA	NA		Weak
Tragelaphus scriptus	Bushbuck	Weak	Strong	NA	NA	NA	NA		Weak
Hippotragus niger	Sable antelope	Weak	Strong	None	High	None	Reacted	Equivalent to control	Moderate
Kobus ellipsiprymnus	Waterbuck	Weak	Strong	None	High	NA	NA		Moderate
Equus quagga burchellii	Burchell's zebra	NA	NA	NA	NA	NA	NA		Strong
Alcelaphus buselaphus	Red hartebeest	NA	NA	NA	NA	NA	NA		Weak
Connochaetes gnou	Black wildebeest	NA	NA	None	low	NA	NA		Strong
Damaliscus lunatus	Common tsessebe	NA	NA	NA	NA	NA	NA		Weak

Damaliscus pygargus phillipsi	Blesbok	NA	NA	None	Medium	NA	NA	Equivalent to control	
Antidorcas marsupialis	Springbok	NA	NA	High	High	None	Reacted		Strong
Hippotragus equinus	Roan antelope	NA	NA	None	Low	NA	NA	Equivalent to control	Moderate
Oryx gazella	Gemsbok	NA	NA	None	Low	NA	NA		Moderate
Tragelaphus strepsiceros	Greater kudu	Weak	Strong	Medium	Medium	None	Reacted	Equivalent to control	No reaction
Bos taurus	Domestic cattle	Weak	Strong	NA	NA	NA	NA	Control	
Capra hircus	Goat	Weak	Strong	NA	NA	NA	NA		
Ovis aries	Sheep	Weak	Strong	NA	NA	NA	NA	Lesser to control	
Damaliscus pygargus	Bontebok							Equivalent to control	

Table S2: SDS-PAGE gel (8%) reagents and volume of separating and stacking gel (Laemmli, 1970)

**Reagents**	**Separating gel (ml)**	**Stacking gel (ml)**
Distilled water	7	2.1
30% Acrylamide	4	0.5
1.5 M Tris (pH 8.8)	3.8	0.380
10% SDS	0.150	0.030
10%APS	0.150	0.030
TEMED	0.009	0.003

Supplementary Methodology 1: Briefly, the microtiter plates (Thermo Scientific™ Pierce 96-well Plates-Corner, USA) were coated overnight with 25 µg/ml of the extracted IgG from the respective species (impala and kudu) as described by Staak*, et al.,* (6). Plates were washed twice with Phosphate Buffered Saline (PBS) supplemented with 0.05% Tween-20 (Thermo Fisher Scientific, Waltham, MA USA) (PBST) using a plate washer (Biorad PW40, Mamesla-Coquette, France). The plates were blocked blocked with PBST supplemented with 5% skimmed milk powder (PBSTM) and then incubated for 1 hour at room temperature. This was followed by washing the plates twice. A 100 µL of the the egg yolk from each chicken was added into the plate with a starting dilution of 1:20 in PBSTM starting from the first column of each plate. This was followed by 30 minutes incubation on a rotatory incubator (Environmental Shaker-Incubator ES-20, Biosan Ltd, Germany). Afterwards, the plates were washed five times and a 100 µL of a 1:10000 dilution of goat anti-chicken horseradish peroxidase conjugate (Invitrogen goat anti-chicken, USA) was added to each well and incubated for 30 minutes on the rotary incubator. This was followed by a wash step and subsequently, the substrate 2,2'-Azinobis[3-ethylbenzothiazoline-6-sulfonic acid]-diammonium salt (ABTS) (Thermo Scientific 1-Step ABTS, USA) was added and incubated in the dark for 45 minutes. The absorbance was read at 405 nm using the plate reader(Biotek Powerwave XS2 reader, Vermont, USA).

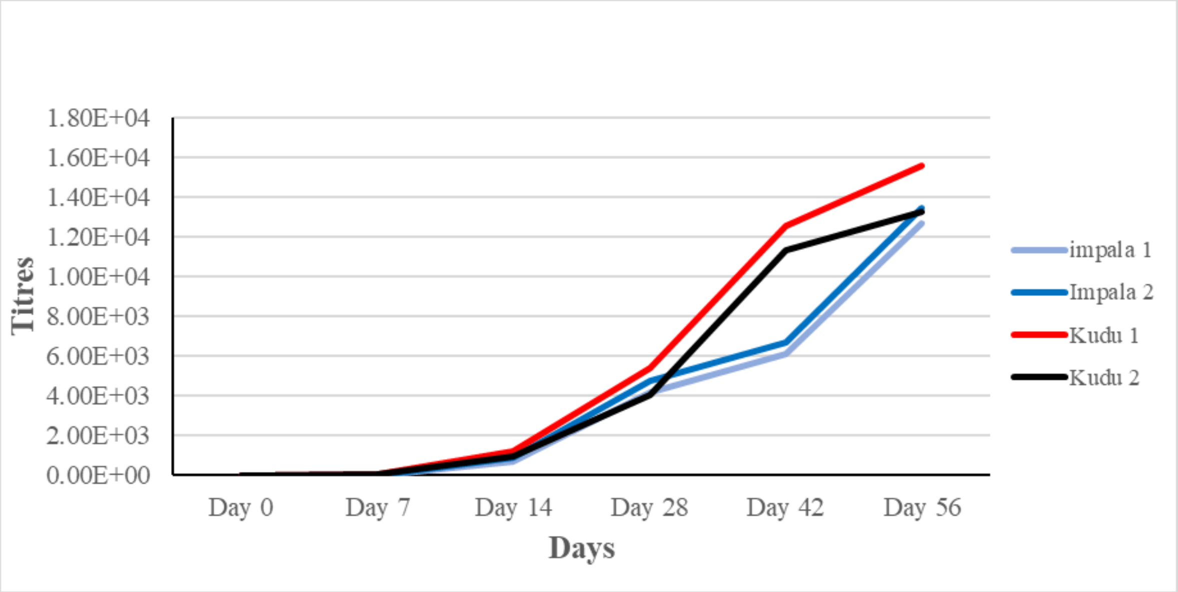

Figure S1: Line chart showing the increase in titres over days of antibodies against impala and kudu Immunoglobulin G (IgG). The first vaccination was given on "Day 0", the second dose was given on "Day 23" and the last dose was given on "Day 42"

Supplementary Methodology 2: A pooled sera for each species (impala and kudu) were coated to microtiter plates with a starting dilution of 1:1000 from column 1 to 11 in coating buffer (bicarbonate buffer) left overnight at 4oC to incubate. This was followed by a blocking step with the blocking buffer (200 µL) containing PBST and 5% skimmed milk powder (PBSTM) and then incubated at room temperature for 1 hour. The developed conjugates were tested against each species with a starting dilution of 1:200 row A to row G. The blanked wells are row H and column 11. The plates were incubated at room temperature for 30 minutes. Subsequently, the plates were washed and after which the ABTS substrate (2,2'-Azinobis [3-ethylbenzothiazoline-6-sulfonic acid]-diammonium salt; Thermo Scientific 1-Step ABTS, USA) was added and allowed in the dark for 45 minutes. The absorbance was read at 405 nm using the plate reader (Biotek Powerwave XS2 reader, Vermont, USA).

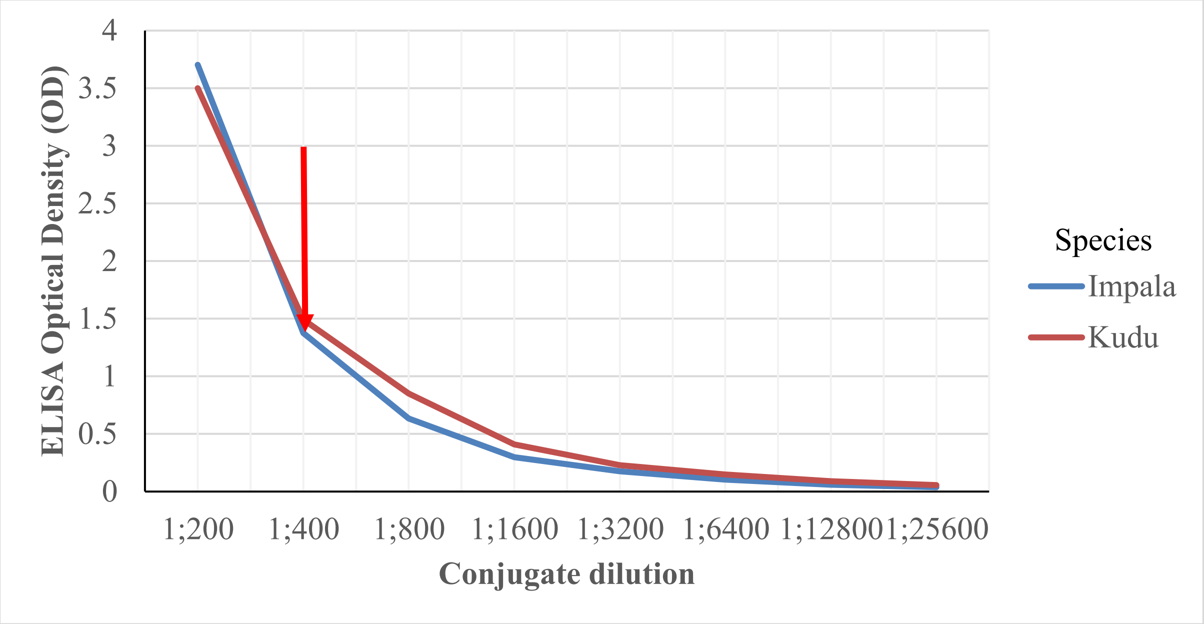

Figure S2: Line graph showing the concentration (Optical Density) of chicken anti-kudu and chicken anti-impala conjugates at different dilutions. The blue line represents anti-impala conjugate while the orange line represents anti-kudu conjugate. The red arrow depicts the optimal dilution (1:400) for the conjugates.

Table S3: Anti-impala summary statistics showing mean OD of PBS and potassium thiocyanate treated wells, mean OD of PBS and chaotrope treated wells for the goat control, percentage reduction in OD, and the mean avidity index and standard deviation (SD) and the p-value of the independent T-test comparing avidity index of each species and the impala control.

**Species**	**Mean OD ± SD PBS**	**Mean OD ± SD chaotrope**	**Mean OD impala Control**	**Mean OD control chaotrope**	**Percentage reduction**	**Mean Avidity index ± SD**	**P- value**
African buffalo	1.64 ± 0.03	1.06 ± 0.02	1.67	1.03	35.18	64.82 ± 1.37	4.58E-10
Black wildebeest	1.37 ± 0.05	0.81 ± 0.05	1.62	1.34	40.50	59.5 ± 1.86	9.77E-11
Blesbok	1.46 ± 0.04	1.07 ± 0.02	1.54	1.15	26.82	73.18 ± 1.62	0.088907
Blue wildebeest	1.8 ± 0.04	1.07 ± 0.04	1.86	1.35	40.30	59.7 ± 1.55	3.10E-12
Bontebok	1.16 ± 0.02	0.82 ± 0.01	1.86	1.35	29.12	70.88 ± 0.87	0.007503
Bushbuck	1.16 ± 0.02	0.78 ± 0.02	1.68	1.18	32.93	67.07 ± 0.39	1.48E-09
Cattle	0.82 ± 0.02	0.34 ± 0.01	1.84	1.35	59.05	40.95 ± 0.9	5.17E-23
Eland	1.26 ± 0.03	0.74 ± 0.02	1.72	1.21	40.94	59.06 ± 0.51	1.07E-15
Gemsbok	1.24 ± 0.02	0.87 ± 0.02	1.86	1.35	29.66	70.34 ± 0.86	0.000329
Giraffe	1.41 ± 0.05	0.74 ± 0.02	1.62	1.34	47.29	52.71 ± 0.52	3.85E-18

Goat	0.94 ± 0.03	0.42 ± 0.01	1.84	1.35	55.11	44.89 ± 0.5	8.84E-20
Impala	1.96 ± 0.03	1.41 ± 0.02	1.96	1.36	27.91	72.09 ± 0.89	NA
Kudu	1.52 ± 0.03	1.01 ± 0.02	1.86	1.35	33.33	66.67 ± 1.23	5.30E-09
Nyala	1.64 ± 0.03	1.13 ± 0.01	1.84	1.35	31.19	68.81 ± 0.4	1.71E-07
Plains zebra	0.36 ± 0.03	0.11 ± 0.01	1.86	1.35	69.45	30.55 ± 1.04	5.32E-24
Red hartebeest	1.42 ± 0.04	0.76 ± 0.02	1.54	1.15	46.44	53.56 ± 1.46	3.41E-15
Roan	1.47 ± 0.02	0.96 ± 0.02	1.72	1.21	34.92	65.08 ± 0.72	7.79E-13
Sable	1.08 ± 0.02	0.76 ± 0.02	1.84	1.35	30.14	69.86 ± 1.81	0.004318
Sheep	0.41 ± 0.05	0.24 ± 0.03	1.68	1.18	41.47	58.53 ± 2.14	4.74E-10
Springbok	1.55 ± 0.02	1.14 ± 0.02	1.85	1.36	26.31	73.69 ± 1.04	0.002179
Tsessebe	1.64 ± 0.03	1.06 ± 0.02	1.67	1.03	35.18	64.82 ± 1.37	4.58E-10
Waterbuck	1.84 ± 0.04	1.02 ± 0.03	1.67	1.03	44.68	55.32 ± 0.29	2.16E-14

Table S4: Anti-kudu summary statistics showing mean OD of PBS and potassium thiocyanate treated wells, mean OD of PBS and chaotrope treated wells for the goat control, percentage reduction in OD, and the mean avidity index and standard deviation (SD) and the p-value of the independent T-test comparing avidity index of each species and the kudu control. Abbreviation such as NA means Not Applicable.

**Species**	**Mean OD ± SD PBS**	**Mean OD ± SD chaotrope**	**Mean OD kudu Control**	**Mean OD control chaotrope**	**Percentage reduction**	**Mean Avidity index ± SD**	**P- value**
African buffalo	1.72 ± 0.06	1.19 ± 0.04	1.83	1.33	30.73	69.27 ± 0.64	2.91E-06
Black wildebeest	1.75 ± 0.03	1.11 ± 0.01	2.47	1.80	36.59	63.41 ± 0.49	2.80E-11
Blesbok	1.76 ± 0.06	1.15 ± 0.03	1.76	1.31	34.37	65.63 ± 0.86	5.77E-11
Blue wildebeest	0.85 ± 0.02	0.52 ± 0.01	1.43	1.09	39.03	60.97 ± 0.71	6.58E-14
Bontebok	0.74 ± 0.02	0.52 ± 0.02	1.46	1.20	30.23	69.77 ± 0.71	2.07E-05
Bushbuck	0.87 ± 0.04	0.61 ± 0.02	1.65	1.21	29.74	70.26 ± 0.72	0.000188
Cattle	0.66 ± 0.03	0.26 ± 0.01	1.65	1.18	60.72	39.28 ± 0.99	4.67E-22
Eland	2.15 ± 0.06	1.53 ± 0.03	2.18	1.59	29.07	70.93 ± 1.38	0.022406
Gemsbok	1.02 ± 0.05	0.81 ± 0.04	1.86	1.34	20.78	78.92 ± 1.18	3.03E-10
Giraffe	1.34 ± 0.04	0.73 ± 0.02	2.47	1.80	45.49	54.51 ± 0.69	1.47E-16
Goat	0.51 ± 0.06	0.2 ± 0.02	1.64	1.18	60.28	39.72 ± 0.87	1.39E-21
Impala	1.61 ± 0.04	1.13 ± 0.02	1.78	1.30	29.80	70.2 ± 1.05	0.000364
Kudu	1.86 ± 0.03	1.34 ± 0.02	1.86	1.35	27.64	72.36 ± 1.13	NA
Nyala	1.85 ± 0.04	1.5 ± 0.03	1.86	1.34	18.68	80.82 ± 1.42	3.76E-11
Plains zebra	0.56 ± 0.02	0.2 ± 0.01	1.43	1.09	64.03	35.97 ± 0.38	1.81E-17

Red hartebeest	1.51 ± 0.05	1 ± 0.03	1.76	1.31	33.77	66.23 ± 0.83	1.63E-10
Roan	2.14 ± 0.06	1.41 ± 0.03	2.18	1.59	34.03	65.97 ± 0.82	8.90E-11
Sable	1.76 ± 0.02	1.12 ± 0.01	1.89	1.67	36.16	63.84 ± 0.69	2.73E-12
Sheep	0.67 ± 0.04	0.45 ± 0.02	1.65	1.21	33.49	66.51 ± 1.72	1.53E-07
Springbok	1.6 ± 0.04	0.96 ± 0.03	1.80	1.31	40.07	59.93 ± 0.46	5.74E-13
Tsessebe	1.32 ± 0.04	0.83 ± 0.02	1.63	1.18	37.55	62.45 ± 0.72	2.08E-13
Waterbuck	1.26 ± 0.02	1.63 ± 0	1.18	1.18	36.28	63.72 ± 0.54	1.43E-11

Table S5: Protein AG summary statistics showing mean OD of PBS and potassium thiocyanate treated wells, mean OD of PBS and chaotrope treated wells for the goat control, percentage reduction in OD, and the mean avidity index and standard deviation (SD) and the p-value of the independent T-test comparing avidity index of each species and the cattle control. Abbreviation such as NA means Not Applicable.

**Species**	**Mean OD ± SD PBS**	**Mean OD ± SD chaotrope**	**Mean OD cattle Control**	**Mean OD control chaotrope**	**Percentage reduction**	**Mean Avidity index ± SD**	**P- value**
African buffalo	3.64 ± 0.05	0.68 ± 0.02	2.46	1.83	81.28	18.72 ± 0.63	1.14E-20
Black wildebeest	2.87 ± 0.06	1.41 ± 0.04	2.26	1.46	50.78	49.22 ± 0.69	2.96E-17
Blesbok	2.49 ± 0.1	1.02 ± 0.06	2.33	1.75	59.00	41 ± 1.04	2.76E-21
Blue wildebeest	2.53 ± 0.05	1.25 ± 0.03	2.66	1.93	50.58	49.42 ± 0.53	1.07E-15
Bontebok	3.36 ± 0.08	0.97 ± 0.02	2.43	1.83	71.20	28.8 ± 0.68	2.63E-20
Bushbuck	1.86 ± 0.03	0.52 ± 0.02	2.12	1.56	72.16	27.84 ± 0.94	7.27E-23
Cattle	2.43 ± 0.04	1.83 ± 0.03	2.43	1.83	24.49	75.51 ± 1.29	NA
Eland	2.53 ± 0.03	0.67 ± 0.01	2.63	1.92	73.59	26.41 ± 0.31	1.39E-16
Gemsbok	3.59 ± 0.02	0.95 ± 0.02	2.43	1.83	73.49	26.51 ± 0.49	2.36E-18
Giraffe	2.4 ± 0.07	1.05 ± 0.03	2.46	1.83	56.50	43.5 ± 0.71	1.88E-18
Goat	2.21 ± 0.04	1.77 ± 0.04	2.43	1.83	20.20	79.8 ± 0.9	2.43E-07
Impala	2.49 ± 0.21	0.53 ± 0.04	2.21	1.77	78.53	21.47 ± 0.66	9.31E-21
Kudu	2.77 ± 0.11	0.42 ± 0.02	2.21	1.77	84.77	15.23 ± 1.1	5.96E-25
Nyala	2.52 ± 0.09	0.85 ± 0.05	3.70	2.74	66.34	33.66 ± 1.06	1.31E-22
Plains zebra	2.66 ± 0.05	1.37 ± 0.03	2.66	1.93	48.67	51.33 ± 0.48	6.22E-15
Red hartebeest	2.19 ± 0.05	0.73 ± 0.04	2.33	1.75	66.62	33.38 ± 1.12	9.05E-23

Roan	2.78 ± 0.13	0.79 ± 0.05	2.63	1.92	71.45	28.55 ± 0.72	8.83E-21
Sable	2.46 ± 0.16	0.35 ± 0.04	2.43	1.83	85.72	14.28 ± 2.15	6.41E-20
Sheep	1.93 ± 0.05	1.05 ± 0.03	2.12	1.56	45.50	54.5 ± 1.45	3.76E-17
Springbok	2.52 ± 0.09	0.85 ± 0.05	3.70	2.74	66.34	33.66 ± 1.06	1.31E-22
Tsessebe	2.81 ± 0.03	0.81 ± 0.03	2.66	1.96	71.09	28.91 ± 1.26	2.41E-23
Waterbuck	1.89 ± 0.06	0.93 ± 0.03	2.66	1.96	50.48	49.52 ± 0.8	4.66E-18

Table S6: Protein G summary statistics showing mean OD of PBS and potassium thiocyanate treated wells, mean OD of PBS and chaotrope treated wells for the goat control, percentage reduction in OD, and the mean avidity index and standard deviation (SD) and the p-value of the independent T-test comparing avidity index of each species and the goat control. Abbreviation such as NA means Not Applicable.

Species	Mean OD ± SD PBS	Mean OD ± SD chaotrope	Mean OD goat Control	Mean OD control chaotrope	Percentage reduction	Mean Avidity index ± SD	P- value
African buffalo	1.3 ± 0.04	0.24 ± 0.01	2.58	1.90	81.19	18.81 ± 0.75	2.85E-24
Black wildebeest	2.45 ± 0.06	1.4 ± 0.03	2.58	1.90	42.76	57.24 ± 0.88	4.11E-16
Blesbok	2.53 ± 0.03	0.66 ± 0.01	2.63	1.93	73.70	26.3 ± 0.59	7.91E-27
Blue wildebeest	1.7 ± 0.02	0.46 ± 0.02	2.14	1.59	73.21	26.79 ± 0.78	5.41E-23
Bontebok	1.64 ± 0.09	0.43 ± 0.03	2.14	1.59	73.46	26.54 ± 1.03	1.77E-19
Bushbuck	1.94 ± 0.02	0.54 ± 0.01	2.16	1.58	72.19	27.81 ± 0.38	1.13E-30
Cattle	1.36 ± 0.03	0.75 ± 0.02	2.14	1.59	45.14	54.86 ± 0.55	8.68E-23
Eland	2.15 ± 0.03	0.98 ± 0.02	2.42	1.75	54.71	45.29 ± 0.82	8.02E-20
Gemsbok	1.52 ± 0.11	0.28 ± 0.02	2.14	1.59	81.76	18.24 ± 1.37	1.48E-17
Giraffe	2.62 ± 0.06	1.16 ± 0.04	2.58	1.90	55.50	44.5 ± 1.03	2.87E-17
Goat	2.14 ± 0.03	1.59 ± 0.02	2.14	1.59	25.47	74.53 ± 0.41	NA
Impala	2.89 ± 0.08	0.68 ± 0.01	2.14	1.59	76.48	23.52 ± 0.56	7.61E-28
Kudu	2.82 ± 0.09	0.66 ± 0.02	2.14	1.59	76.39	23.61 ± 0.99	3.19E-20
Nyala	1.61 ± 0.08	0.47 ± 0.04	2.16	1.58	70.94	29.06 ± 2.45	3.85E-13
Plains zebra	1.08 ± 0.09	0.19 ± 0.01	2.14	1.59	82.01	17.99 ± 1.09	1.14E-19
Red hartebeest	2.54 ± 0.04	0.76 ± 0.03	2.63	1.93	70.20	29.8 ± 1.01	3.36E-17
Roan	2.06 ± 0.03	1.02 ± 0.02	2.42	1.75	50.80	49.2 ± 0.23	1.66E-23

Sable	1.16 ± 0.08	0.19 ± 0.01	2.16	1.58	83.42	16.58 ± 0.71	3.17E-25
Sheep	2.15 ± 0.03	1.28 ± 0.01	2.16	1.58	40.32	59.68 ± 0.26	8.46E-22
Springbok	1.56 ± 0.03	0.55 ± 0.01	2.14	1.59	64.62	35.38 ± 0.87	1.66E-20
Tsessebe	2.41 ± 0.03	1.21 ± 0.02	2.47	1.85	49.62	50.38 ± 0.65	1.05E-21
Waterbuck	2.32 ± 0.08	0.86 ± 0.03	2.47	1.85	62.86	37.14 ± 0.47	6.05E-28

References

1. Kelly PJ, Tagwira M, Matthewman L, Mason PR, Wright EP. Reactions of sera from laboratory, domestic and wild animals in Africa with protein A and a recombinant chimeric protein AG. Comparative immunology, microbiology and infectious diseases. 1993;16(4):299-305.
2. Stöbel K, Schönberg A, Staak C. A new non-species dependent ELISA for detection of antibodies to *Borrelia burgdorferi* s. l. in zoo animals. International Journal of Medical Microbiology: Supplement 33. 2002;291(Supplement 33):88-99.
3. Feir D, Lau C, Junge R. Protein A and protein G in the diagnosis of diseases in zoo animals. Transactions of the Missouri Academy of Science. 1993;27:9-14.
4. Kramsky JA, Manning EJ, Collins MT. Protein G Binding to Enriched Serum Immunoglobulin from Nondomestic Hoofstock Species. Journal of Veterinary Diagnostic Investigation. 2003;15(3):253-61.
5. Smit SA. Evaluation of anti-bovine, anti-equine and recombinant protein A/G horseradish peroxidase conjugates for cross reactivity to wildlife serum antibodies using ELISA: University of Pretoria; 2017.
6. Staak C, Salchow F, Denzin N. Practical serology : from the basics to the testing. München: Urban & Vogel; 2001.
